# Endocrine dysfunctions in patients with beta thalassemia major: a single-center retrospective study from Basrah

**DOI:** 10.12688/f1000research.182800.2

**Published:** 2026-09-15

**Authors:** Haider Alidrisi, Hussein Nwayyir, Mahmood Altemimi, Nassar Alibrahim, Ola Hamid, Israa Abdulbari, Nargis Noman, Abbas Mansour

**Affiliations:** 1 Faiha Specialized Diabetes, Endocrine, and Metabolism Center, University of Basrah College of Medicine, Basrah, Basra Governorate, Iraq; 2 Thi‑Qar Specialized Diabetes, Endocrine and Metabolism Center, University of Thi-Qar, Nasiriyah, Dhi Qar Governorate, Iraq

**Keywords:** beta thalassemia major, growth hormone deficiency, diabetes mellitus, hypogonadism, hypothyroidism, hypoparathyroidism, adrenal insufficiency, iron overload.

## Abstract

**Background:**

The management of patients with beta thalassemia major (BTH) involves regular blood transfusions which are associated with iron overload. This may result in various endocrine dysfunctions (EDs). This study aimed to assess the obesrved frequency of different EDs in patients with transfusion-dependent BTH.

**Method:**

A retrospective study that included 496 patients with BTH who were referred from the Basra Center for hereditary blood diseases to Faiha Specialized Diabetes, Endocrine and Metabolsim Center for endocrine-related complaints and underwent detailed endocrine assessments from Jan 2010 to Dec 2023.

**Results:**

The mean presentation age for endocrine evaluation was 13.6 ± 6.5 years with no difference between male and female. The commonest clinical presentations of BTH patients in our center were short stature 65.5%, amenorrhea 16.4, hyperglycemia 13.7%, delayed puberty 13.3, and hypocalcemia 5.2%. The proportion patients with ED was 16.8%. The most frequent EDs were growth hormone deficiency (GHD) 6.0%, diabetes mellitus (DM) 4.9%, hypogonadism 4.5%, hypothyroidism 3.2%. Within those patients, 78.7% had single ED, 16.5% had two EDs and 4.8% had three or more EDs.

**Conclusion:**

EDs were observed among patients with BTH. The commonest EDs were GHD, DM, and hypogonadism. We recommend regular and timely surveillance using both clinical and hormonal assessment in patients with BTH.

## Introduction

Beta thalassemia major (BTH) is an inherited impairment of hemoglobin production. The last estimated total number of registered cases of thalassemia in Iraq is 13390 giving a prevalence of 3.4/10000. ^1^ A study in Iraq showed that about 66.0% of the patients were under 15 years of age, 78.8% of whom reported related parents (consanguineous marriage). Thalassemia represented 75% of all hemoglobinopathies in Iraq. The highest prevalence was registered in Basrah province, with BTH representing 67% of all types of thalassemia. ^2^

In BTH, defective production from disabling point mutations causes no (β0) or reduced (β–) beta chain production. Homozygotes (BTH) either are unable to synthesize hemoglobin A or produce very little after the first 4–6 months of life. This results in profound transfusion-dependent hypochromic anemia. ^3^

In patients with BTH, the recommended treatment involves regular red blood cell transfusions throughout life, every 2–5 weeks to maintain pre-transfusion Hb above 9–10.5 g/dl. ^4^ The transfusion regimen promotes proper growth, allows normal physical activities, and adequately inhibits ineffective erythropoiesis. ^9^ However, this requires to balancing consequences from anemia against complications of iron overload (IO). ^5^ In the case of IO, there will be saturation of transferrin and non-transferrin-bound iron (NTBI) species circulating in the plasma. Unbound iron within cells or plasma redox cycle between Fe2+ and Fe3+, thereby generating reactive oxygen species. This led to lipid peroxidation, which led to the generation of both unsaturated (malondialdehyde and hydrodynamical) and saturated (hexanal) aldehydes. Both have been implicated in cytotoxicity and cell death. ^6^ ^,^ ^7^

The endocrine system is particularly sensitive to IO. The spectrum of endocrine dysfunctions (EDs) in BTH can be severe and put more challenges to the already complicated health in BTH patients. ^8^ The pattern of EDs may involve growth hormone deficiency (GHD), delayed puberty and gonadal dysfunctions, primary or secondary hypothyroidism, primary or secondary adrenal insufficiency (AI), primary hypoparathyroidism (hypoPTH), and diabetes mellitus (DM). ^9^ That is why early and timely detection of these endocrine complications is valuable for their effective management.

The study aimed to assess the observed frequency of different EDs among patients with transfusion-dependent BTH in Basrah.

## Methods

A retrospective study with database retrieval was conducted in at Faiha Specialized Diabetes, Endocrine, and Metabolism Center (FDEMC). The patients with transfusion-dependent BTH who were referred to the center for endocrine evaluation from Jan 2010 to Dec 2023 were included in the analysis that was done from Oct 2023 to Feb 2024. The Basra Center for hereditary blood diseases have a registry of the patients with various types of hemoglobinopathies including BTH. Until the end of 2023, 1365 patients with BTH were registered, received treatment and periodic clinical evaluation. ^10^ We included patients who were diagnosed with transfusion-dependent BTH and referred to the center for endocrine-related complaints in the form of short stature (ST), hyperglycemia, amenorrhea, delayed puberty, and hypocalcemia. Patients with incomplete clinical and or laboratory data were excluded for this study. The study was approved by the Iraqi Ministry of Health and the Institutional Review Board of the Faiha Specialized Diabetes, Endocrine, and Metabolism Center (FDEMC) of the Basrah Health Directorate. Due to the retrospective design and use of anonymized patient data, the requirement for informed consent was waived by the Institutional Board Review of the FDEMC.

From 2010 to 2023, from total 1365, 563 patients with transfusion-dependent BTH were referred from Basra Center of hereditary blood diseases to FDEMC for endocrine evaluations. Only 496 patients had complete data and were included in the final analysis, (277 male and 219 female, and age range 1–42 years at the time of evaluation). Through a similar timeframe (2010–2023), 11229 patients with no BTH were diagnosed with a comparable endocrine disorder. The patients were in the form of type 1 DM 4100 patients, GHD 1566 patients, hypogonadism 517 patients (hypogonadotropic hypogogonadism (hypoG H) 416 and hypergonadotropic hypogonadism (hyperG H) 101), hypothyroidism 4058 patients, AI 737 patients, and hypoPTH 251 patients.

### Endocrine assessment

#### Hypogonadism

Female patients were considered as primarily amenorrhoeic by the absence of menses at fifteen-year-old age or thirteen-year-old age with absent secondary sexual characteristics. Secondary amenorrhea was defined by an absence of menses for more than three months in females who previously had regular menstrual cycles or six months in girls or women who had irregular menses. For every female, a pelvic ultrasound examination was done to assess of presence of the uterus and ovaries. A specialized endocrinologist examined the patients for the degree of breast maturation in female patients and testicular volume in male patients. The patients were considered as having delayed puberty by the absence of breast development in a twelve-year-old girl and testicular enlargement in a fourteen-year-old boy.

In females, presented with delayed puberty and or amenorrhea (primary or secondary), a Follicular stimulating hormone (FSH) level above normal indicated hyperG H and was followed by karyotype assessment when available. In males, a total testosterone (TT) level below the reference range was followed by a repeated confirmatory low TT level. If the luteinizing hormone (LH) level was above normal, hyperG H was diagnosed and was followed by karyotype when available. While a normal or low FSH and LH levels indicated hypoG H in both sexes.

#### Growth hormone deficiency

For every patient with bare feet and light clothes, the height (HT) and body weight (BWT) were measured using a stadiometer and a scale respectively. The patients were considered ST if the Length (less than two years old) or HT (two and above years old) is more than 2 standard deviations (SD) below the mean (Z-score < −2), which corresponds to an HT that is <2.3rd percentile. Bone age was determined in all patients before hormonal evaluations.

For each child presented with ST as defined by age, gender and mid-parental HT, a growth hormone (GH) stimulation test with glucagon was done. The tests were done in the early morning after eight hours of overnight fasting. GH was measured at baseline and every 30 minutes for the next two hours after subcutaneous injection of 1 milligram (0.5 milligram if BWT was less than 25 kilograms). A baseline GH of (7 ng/mL [21.02 mIU/L]) or more excluded GHD. If baseline GH was less than (7 ng/mL [21.02 mIU/L]), failure of the GH level to exceed (10 ng/mL [30.0 mIU/L]) through post glucagon injection indicates GHD. Further assessment included measurements of anti-tissue transglutaminase immunoglobulin A antibodies (tTg-IgA Ab), thyroid-stimulating hormone (TSH), and free thyroxine (fT4) to exclude celiac disease and hypothyroidism.

#### Diabetes mellitus

DM was diagnosed based on fasting serum glucose above or equal to (126 mg/dL [7.0 mmol/L]) (two occasions), or non-fasting blood glucose above or equal to (200 mg/dL [11.1 mmol/L]) (two occasions).

#### Hypothyroidism

Both serum TSH and fT4 were used for the diagnosis. A TSH level above 10 mU/l and low fT4 indicated primary hypothyroidism. A TSH level above normal and normal fT4 indicates subclinical primary hypothyroidism. While a low, normal, or up to 10 mU/l TSH level and low fT4 indicated central hypothyroidism.

#### Primary hypoparathyroidism

Patients were diagnosed with primary hypoPTH by the finding of low calcium on two occasions and normal or low parathormone (PTH) levels.

#### Adrenal insufficiency

Patients were diagnosed with AI based on a serum cortisol level less than 137.94 nmol/L (5 Mg/dl). In the case of normal or low plasma adrenocorticotropic hormone (ACTH), the patients were diagnosed with secondary AI. While the patients were diagnosed with primary AI if they had a high plasma ACTH level. Dynamic testing using ACTH stimulation test was performed only when the morning serum cortisol was more than 137.95 nmol/L (5 Mg/L) and less than 275.88 nmol/L (10 Mg/dl). If cortisol failed to increase more than 496.58 nmol/L (18 Mg/dl) with ACTH stimulation we considered the patient as having AI.

### Laboratory data

After eight hours of overnight fasting, ten milliliters were drawn from each patient, seven milliliters put in a clot activator tube for serum for the measurements of GH, TSH, fT4, FSH, LH, estradiol (E2), prolactin (PRL), TT, cortisol, PTH, tTg-IgA Ab, glucose, 25 hydroxy vitamin D (25-OH D), calcium (Ca), albumin, and phosphorus (PO4). The remaining three milliliters were put in an ethylene diamine tetra acetic acid tube for plasma ACTH.

The Fully automated chemiluminescence immunoassay kits Cobas e411 analyzer series/Roche Diagnostics, Germany, was used for the measurements of GH (reference value 0.7–50 ng/mL, [2.10–150.15 mIU/L]), TSH (reference value 0.27–4.2 μIU/mL), fT4 (reference value 0.93–1.7 ng/dL [11.97–21.88 pmol/L]), FSH (reference value for male 1–13 mIU/mL, and Female 2–12 mIU/mL), LH (reference value for male 1–9 mIU/mL, and female 1–18 mIU/mL), E2 (reference value for female 15–30 pg/mL [55.47–110.94 pmol/L]), PRL (reference value for male 4–23 ng/mL (85.10–489.36 mIU/L), and for female 4–30 ng/mL (85.10–638.29 mIU/L)), TT (reference value for male 265 to 1200 ng/dL [9.20–41.64 nmol/L]), ACTH reference value 10–60 pg/ml [2.20–13.20 pmol/L]), cortisol (reference value 5–25 μg/dL [137.94–689.70 nmol/L]), PTH (reference value 15–65 pg/mL [1.59–6.89 pmol/L]), and 25-OH D (reference value 30 to 80 ng/dL [74.88–199.68 nmol/L]). Measurements of serum glucose, Ca, PO4, and albumin were done by Cobas, INTEGRA 400 PLUS, Roche Company, Switzerland. The tTg-IgA Ab was measured using the ALESKU.DIAGNOSTICS GmbH tTg-A new generation, Germany.

### Statistical analysis

The Statistical Package for the Social Sciences (SPSS), version 26.0 (IBM Corp., Armonk, NY, USA) was used for data analysis. Qualitative variables were summarized as numbers (N) and percentages (%) and continuous variables were summarized as mean ± standard deviation (M ± SD). Correlations between qualitative variables was done using Chi-Square test and Fisher Exact test. And correlations between qualitative and quantitative variables was done by the independent student t test. For all of comparisons, a P value of less than 0.05 was considered as a statistically significant. We considered the overall cohort of 1365 (710 males and 655 females) patients with BTH who being registered in the Basra Center for hereditary blood diseases as the denominator for the observed proportions calculations.

## Results

Table 1 summarizes the general anthropometric features of the study patients. It showed a mean age of presentation of 13.6 ± 6.5 years old (median 13 years old).

**Table 1.** Baseline anthropometric characteristics of The BTH patients (N 496).

Variable	Male (N 277)	Female (N 219)	Total (N 496)
Age (years), mean ± SD	13.7 ± 6.3	13.5 ± 6.7	13.6 ± 6.5
Age (years), median	13.0	12.0	13.0
BWT (Kg), mean ± SD	30.2 ± 11.7	30.9 ± 13.5	30.5 ± 12.6
HT (cm), mean ± SD	133.9 ± 20.7	131.4 ± 20.9	132.8 ± 20.4
BMI (kg/m2), mean ± SD	16.0 ± 2.4	17.0 ± 3.8	16.5 ± 3.1

Abbreviations: BTH, beta thalassemia major; BWT, body weight; kg, kilogram; HT, height; BMI, body mass index.

The commonest clinical presentations of BTH were ST (65.5%), followed by amenorrhea (16.4), hyperglycemia (13.7%), delayed puberty (13.3), and hypocalcemia (5.2%). As shown in Figure 1.

**Figure 1.**
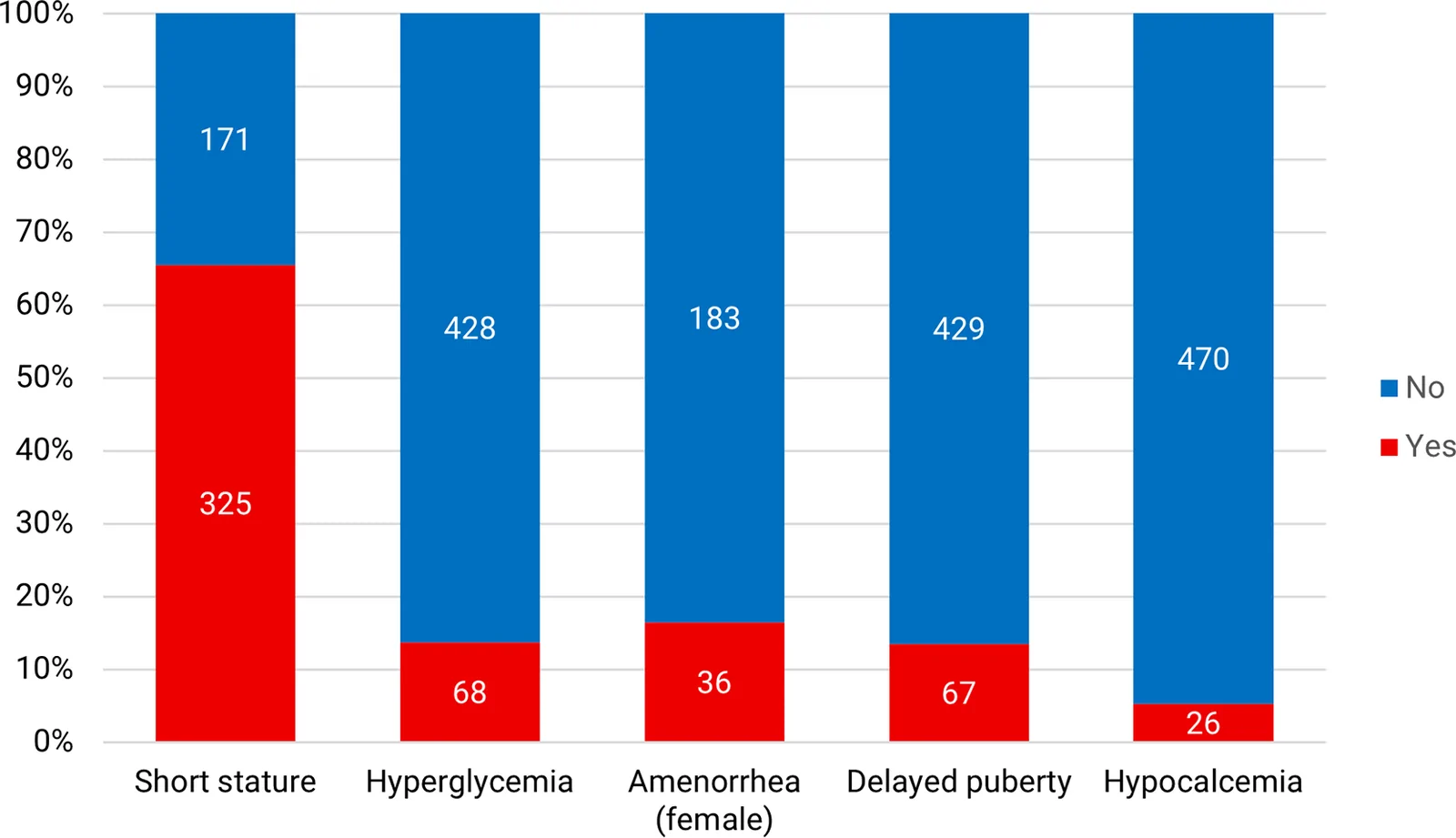
The clinical endocrine presentations of the patients with transfusion-dependent BTH referred to the center (N 496).

In patients with BTH (N 1365), 230 (16.8%) patients had any ED. The most frequent ED was GHD (6.0%), followed by DM (4.9%), HypoG H (4.5%), and hypothyroidism (3.2%). While as a fraction from all EDs diagnosis, patients with BTH were presented in (12.5, 9.3, 5, and 4.7%) of all HypoG H, hypoPTH, GHD, and HyperG H respectively. As shown in Table 2.

**Table 2.** Observed frequencies and percentages of various EDs in transfusion-dependent transfusion-dependent BTH.

ED	N of EDs in BTH	% within referred BTH (N 496)	% within total BTH (N 1365)	% within total patients ^d^
Any ED	230	46.3	16.8	2
GHD	83	25.5 ^a^	6.0	5
DM	68	13.7	4.9	1.6
HypoG H	62	12.5	4.5	12.9
HyperG H	5	1.0	0.4	4.7
Hypothyroidism	44 ^b^	8.9	3.2	1.0
HypoPTH	26	5.2	1.9	9.3
AI	4 ^c^	0.8	0.3	0.5

Abbreviations: BTH, beta thalassemia major; ED, endocrine dysfunction; GHD, growth hormone deficiency; DM, diabetes mellitus; HypoG H, hypogonadotropic hypogonadism; HyperG H, hypergonadotropic hypogonadism; hypoPTH, hypoparathyroidism; AI, adrenal insufficiency.

^a^ From total 325 GH testing.

^b^ All was subclinical hypothyroidism except one with central hypothyroidism.

^c^ Secondary adrenal insufficiency.

^d^ Percentages with total EDs of various causes diagnosed in the center.

The patients were diagnosed with a single ED in 181 (78.7%) out of 230 patients with any ED. Two EDs were diagnosed in 38 (16.5%) and the commonest combinations were in the form of hypogonadism with either DM, GHD or hypothyroidism. Further 11 patients (4.8%) had three or more EDs. As shown in Figure 2.

**Figure 2.**
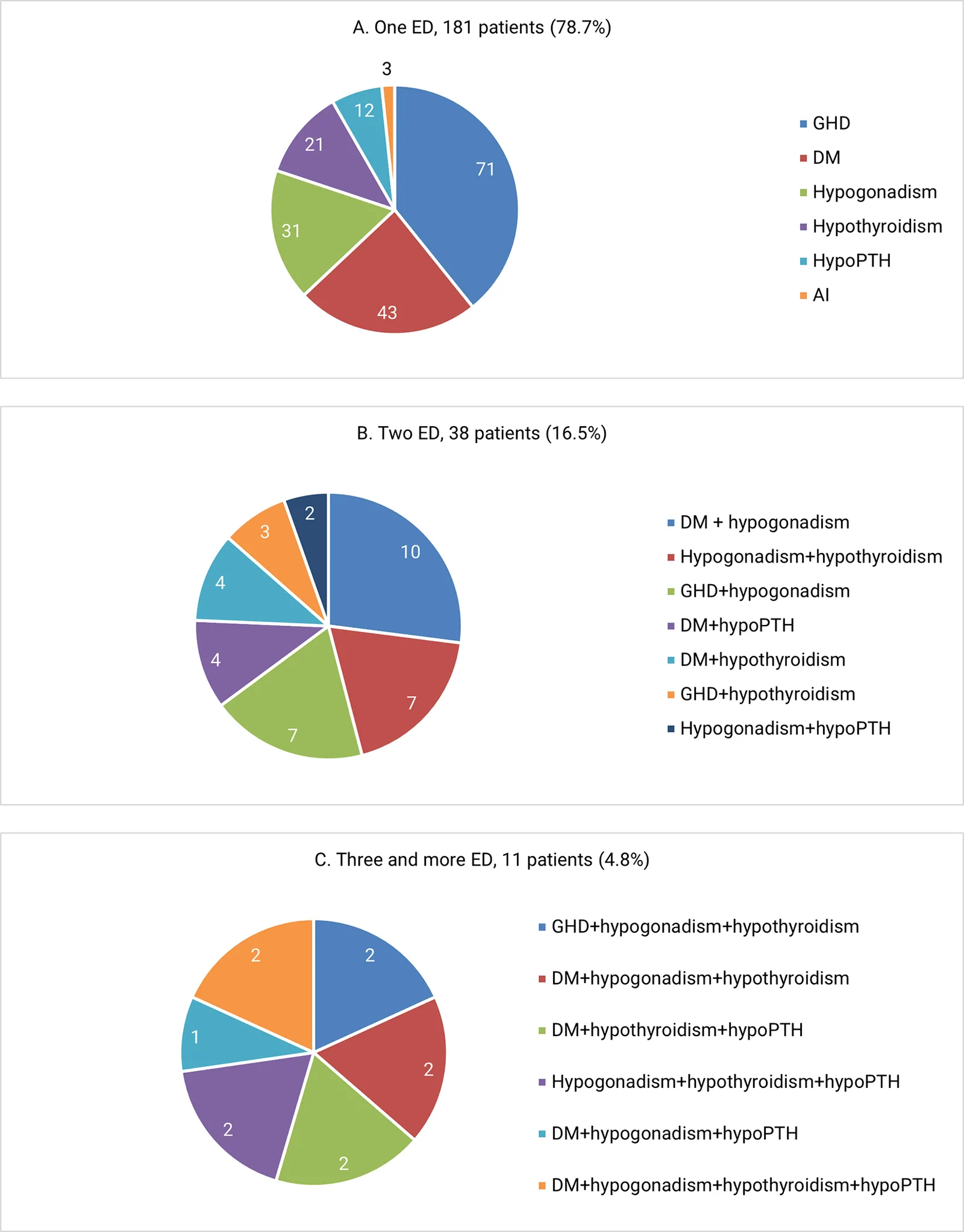
Description of the EDs in the patients with transfusion-dependent BTH (N 230). (A) patients with one ED (B) patients with two ED (C) patients with three or more ED. Abbreviations: ED, endocrine dysfunction; GHD, growth hormone deficiency; DM, diabetes mellitus; hypoPTH, hypoparathyroidism; AI, adrenal insufficiency.

In patients with BTH, short stature as a clinical presentation was significantly more frequent in males 72.6% versus females 56.6%, (P = 0.0002) Other conditions in the form of GHD, DM, HypoG H, HyperG H, hypothyroidism, hypoPTH, and AI did not correlate with gender. With both age and gender-based comparisons within various EDs, males with GHD were significantly older as compared to females (P = 0.02). Further males versus females age comparison did not show a significant difference within DM, HypoG H, HyperG H, hypothyroidism, hypoPTH, and AI. Within either male or female BTH patients, short stature presented younger, while DM and HypoG H presented in older. However, within male patients, hypoPTH also had a significantly older age at diagnosis. Furthermore, in females, HyperG H, hypothyroidism, and AI also had older age at diagnosis. As shown in Table 3.

**Table 3.** Age and gender-based comparisons within various endocrine disorders in patients with BTH.

	Short stature		GHD		DM		HypoG H		HyperG H		Hypothyroidism		HypoPTH		AI	
	Yes	No	Yes	No	Yes	No	Yes	No	Yes	No	Yes	No	Yes	No	Yes ^b^	No
Male N (%)	201 (72.6)	76 (27.4)	48 (23.9)	153 (76.1)	35 (12.6)	242 (87.4)	28 (10.1)	249 (89.9)	2 (0.7)	275 (99.3)	28 ^a^ (10.1)	249 (89.9)	12 (4.3)	265 (95.7)	2 (0.7)	275 (99.3)
Female N (%)	124 (56.6)	95 (43.4)	35 (28.2)	89 (71.8)	33 (15.1)	186 (84.9)	34 (15.5)	185 (84.5)	3 (1.4)	216 (98.6)	16 (7.3)	203 (92.7)	14 (6.4)	205 (93.6)	2 (0.9)	217 (99.1)
P value	0.0002		0.3		0.4		0.07		0.6 ^c^		0.2		0.4		1.0 ^c^	
Male age M ± SD	12.2 ± 3.9	17.5 ± 9.3	11.8 ± 3.0	12.3 ± 4.1	20.6 ± 8.0	12.7 ± 5.4	19.5 ± 6.1	13.0 ± 6.0	22.0 ± 5.6	13.6 ± 6.3	14.9 ± 6.5	13.5 ± 6.3	19.1 ± 6.7	13.4 ± 6.2	10.0 ± 14.1	13.7 ± 6.3
Male age median	13.0	17.0	12.0	13.0	20.0	20.0	18.0	13.0	22.0	13.0	15.0	13.0	20.5	13.0	10.0	13.0
P value within males	<0.001		0.4		<0.001		<0.001		0.06		0.2		0.002		0.4	
Female age M ± SD	11.2 ± 4.1	16.4 ± 8.2	10.8 ± 3.4	11.4 ± 4.3	18.6 ± 7.0	12.6 ± 6.2	17.0 ± 5.8	12.8 ± 6.6	23.6 ± 8.0	13.3 ± 6.6	16.7 ± 8.2	13.2 ± 6.5	16.2 ± 6.0	13.3 ± 6.7	32.0 ± 1.0	13.3 ± 6.5
Female age median	12.0	15.0	10.0	12.0	17.0	12.0	16.0	12.0	19.0	12.0	17.0	12.0	16.5	12.0	32	12
P value within female	<0.001		0.4		<0.001		0.01		0.008		0.045		0.1		<0.001	
P value Male vs female	0.7		0.02		0.7		0.7		0.7		0.7		0.7		0.7	

Abbreviations: BTH, beta thalassemia major; M, mean; SD, standard deviation; BWT, body weight; kg, kilogram; HT, height; BMI, body mass index; GHD, growth hormone deficiency; DM, diabetes mellitus; HypoG H, hypogonadotropic hypogonadism; HyperG H, hypergonadotropic hypogonadism; hypoPTH, hypoparathyroidism; AI, adrenal insufficiency.

^a^ One patient with central hypothyroidism.

^b^ Presented with secondary adrenal insufficiency.

^c^ Fisher Exact p value.

## Discussion

Iron accumulation is a substantial problem in transfusion-dependent adult BTH patients. As a result, endocrine and metabolic disorders are common, but past research reports various prevalence figures. A key factor in the variability of reported prevalences is the differences among patient groups (referral, blood transfusion, iron chelation) included in various studies.

The median age for endocrine presentations in the present study was 13 years old. This presented an average of three years later presentation as compared to studies of Safdar et al. and Ejaz et al. ^11^ ^,^ ^12^ A later referral may explain these age differences.

The EDs that were diagnosed in our study were GHD, DM, hypogonadism, hypothyroidism, hypoPTH, and AI in a descending prevalences. The proportion of either of these EDs varies between studies based on the studies population and the criteria of diagnosis. ^8^ These EDs were presented most singly, or as two EDs, or less commonly as three or more EDs in combination. Additionally, we found lower frequencies of these EDs in our study as compared to other studies. In a retrospective single-center study, 54% had at least one ED, 38.9% had two EDs, and 11.1% had three EDs or more. ^13^

In our study, the commonest clinical presentation was ST, and GHD was the commonest endocrine diagnosis. GHD was presented at a younger age in females. These findings are consistent with a study that was done in Iran in 2006. ^14^ Other results from a meta-analysis in 2021, the prevalence of ST and GHD were 48% and 25% respectively. ^15^ The pathogenesis of growth retardation is multifactorial. Chronic cellular hypoxemia, pituitary iron toxicity, hepatic dysfunction, pubertal failure, and other endocrine dysfunction like hypothyroidism result in growth retardation in patients with BTH. ^8^ ^,^ ^16^ ^,^ ^17^

DM was the second most common ED in our study and presented with a higher median age. Thus, hyperglycemia in BTH is considered a dynamic complication over time due to chronic and progressive IO, hypoxic, inflammatory, and oxidative adverse effects on both pancreatic and hepatic functions. ^18^ In a meta-analysis of 44 studies with a focus on DM in BTH, the rates of DM, impaired fasting glucose, and impaired glucose tolerance were 6.5%, 17.2%, and 12.4% respectively. ^19^

Amenorrhea in females and delayed puberty in both genders were presented as an ED in our study. Hypogonadism was diagnosed in 5.0% and almost always hypoG H. It represents 17.6% of all cases of hypogonadism in the center. Tat et al. had reported a higher prevalence of hypogonadism of 22%. ^13^ More higher prevalences were found by Moayeri et al. ^14^ and Yaghobi et al. ^20^ of (69% and 44% respectively). Furthermore, in the last study, hypogonadism was more common at an age younger than 15 years old. While in the present study, hypogonadism was diagnosed at a significantly higher age. Even higher prevalence of hypogonadism of 82% was found in a study by Ehsan et al. ^21^ The higher median inclusion age of 17 years in the last study may explain such a higher prevalence due to longer period of exposure to IO.

Hypothyroidism prevalence was 3.2%. Most authors suggested that subclinical is the most frequent form, with slight male excess in prevalence contrary to the usual female excess of hypothyroidism in non-BTH patients. ^22^ ^,^ ^23^ Baghersalimi et al. had identified hypothyroidism rate of 10%, with a mean age at diagnosis about 15 years old and none of the cases were central nor overt hypothyroidism. ^24^ Other studies have pointed to a lower prevalence of 8.3% and a higher prevalence of 30% with no age difference in diagnosis. ^20^ ^,^ ^21^

HypoPTH was less commonly observed within BTH patients in present study, but it represented a significant fraction of 9.3% within all cases of hypoPTH of varying causes. That is why BTH could be considered an important cause of hypoPTH in general. Our data suggested a lower prevalence rate as compared to previous studies in which hypoPTH was found in rates of 6.6%, 13.2%, 22%, and 40%. ^20^ ^,^ ^21^ ^,^ ^25^ ^,^ ^26^ These prevalence rates variability may be explained by differences age of inclusion and populations in these studies.

The least common ED in the present study was secondary AI. Most similar studies considered AI as the rarest form of BTH-related EDs. ^8^ In other two studies in which dynamic testing using ACTH stimulation test was used, the rates of diagnosis of AI were 20% and 32%. ^27^ ^,^ ^28^ The frequent non-reliance on dynamic testing for AI diagnosis might underestimate the actual prevalence of this life-threatening endocrine complication of BTH.

The coexistence of two or more endocrine dysfunctions may represent cumulative multisystem endocrine injury related to prolonged IO, chronic hypoxia, oxidative stress, and differences in transfusion or chelation exposure. However, the small number of patients with combined EDs—particularly those with three or more—precluded reliable subgroup comparisons. ^8^ ^,^ ^9^

The study has limitations. First, it was a retrospective and single-center study. Second, it involved patients with BTH that were referred from a blood disease center for probable EDs, which might result in either bias in the observation of these EDs or underestimate the exact frequency due to lack of routine referral and endocrine assessment. Third, dynamic testing for AI was not performed in all patients and might be responsible for its low diagnosis rate. Finally, the study did not include analysis for correlations of the IO indices, adequacy of iron chelation therapy, and transfusion requirement with EDs development.

In conclusion, EDs were observed in patients with transfusion-dependent BTH, and one-fifth of them had combination EDs. The commonest EDs were GHD, DM, and hypogonadism. BTH presented as common cause of hypogonadism and hypoPTH among other causes in our center. Our data suggest lower diagnosis rates of EDs as compared to other studies, indicating underdiagnosis and under referral. We recommend regular and timely surveillance using both clinical and hormonal assessment in patients with BTH.

## Ethical considerations

The Iraqi Ministry of Health and the Institutional Review Board of the FDEMC of the Basrah Health Directorate approved the study (Reference number 56/12/23) in (10/09/2023).

## Data Availability

The data that support the findings of this study are openly available in Figshare: https://doi.org/10.6084/m9.figshare.32301165 (Alidrisi HA et al., 2026) under a CC BY 4.0 license. ^29^
