# Supplementary material for: Endocrine dysfunctions in patients with beta thalassemia major: a single-center retrospective study from Basrah
Source: F1000Res. 2026 Sep 15;15:978. Originally published 2026 Jun 18. [Version 2] doi: 10.12688/f1000research.182800.2 (PMC13639352; doi:10.12688/f1000research.182800.2)
Supplement: Peer review history

# Reviewer report: Reviewer response for version 2

**DOI:** 10.5256/f1000research.210016.r522623

**Published:** 2026-10-05

**Authors:** Anwer Faisal

**Affiliations:** 1 Mustansiriyah University, Baghdad, Iraq

This manuscript presents a valuable retrospective, single-center study addressing the frequency of endocrine dysfunctions (EDs) among patients with transfusion-dependent Beta Thalassemia (BTH) in Basrah, Iraq, over a 13-year period.

 Some questions need to be cleared from the further 

 1. With iron toxicity being the major pathophysiological cause of endocrine dysfunctions (EDs) in thalassemia, can one obtain information regarding serum ferritin levels or compliance with iron chelation therapy from the medical records and correlate this information with the onset of EDs?

 2.You said that dynamic ACTH stimulation testing wasn't routinely done and that Adrenal Insufficiency was the least common dysfunction. Do you think it should be a routine test to be done to prevent the loss of potentially life-threatening cases of secondary AI?

 3. What is your specific plan for endocrine surveillance in the hereditary blood diseases centre in Basrah based on your observations during these 13 years? In particular, what are the routine hormonal tests and when should they start?

 4.Without iron overload indices, how can we be sure that these endocrine disorders were caused by iron overload due to thalassemia and not other genetic or environmental factors?

Is the work clearly and accurately presented and does it cite the current literature?

Partly

If applicable, is the statistical analysis and its interpretation appropriate?

I cannot comment. A qualified statistician is required.

Are all the source data underlying the results available to ensure full reproducibility?

Yes

Is the study design appropriate and is the work technically sound?

Yes

Are the conclusions drawn adequately supported by the results?

Yes

Are sufficient details of methods and analysis provided to allow replication by others?

Yes

Reviewer Expertise:

microbiology

I confirm that I have read this submission and believe that I have an appropriate level of expertise to confirm that it is of an acceptable scientific standard, however I have significant reservations, as outlined above.

# Reviewer report: Reviewer response for version 1

**DOI:** 10.5256/f1000research.201773.r513498

**Published:** 2026-08-31

**Authors:** Pradana Zaky Romadhon, Esthiningrum Dewi Agustin

**Affiliations:** 1 Airlangga University, Surabaya, Indonesia

This is an interesting and eye-awakening study focusing on endocrine dysfunctions in patients with beta thalassemia. However, there are several questions and points which need to be clarified by the authors: 

1. Did the authors include both transfusion-dependent and non-transfusion-dependent beta thalassemia? 
2. What were the most frequent chief complaints of the patients during the data collection? 
3. Since only 496 out of 1365 referred BTH patients that had complete data, we think that mentioning prevalence (%) within total BTH on table 2 is unnecessary. 
4. What is the significant difference in clinical presentation among patients with single ED, double EDs, or triple EDs, based on observation? And what makes patients experience double or triple EDs at the same time? We suggest authors elaborate on the discussion. 

Is the work clearly and accurately presented and does it cite the current literature?

Yes

If applicable, is the statistical analysis and its interpretation appropriate?

Yes

Are all the source data underlying the results available to ensure full reproducibility?

Yes

Is the study design appropriate and is the work technically sound?

Partly

Are the conclusions drawn adequately supported by the results?

Yes

Are sufficient details of methods and analysis provided to allow replication by others?

Yes

Reviewer Expertise:

Hematology, Clinical Oncology

We confirm that we have read this submission and believe that we have an appropriate level of expertise to confirm that it is of an acceptable scientific standard, however we have significant reservations, as outlined above.

# Response

**Published:** 2026-09-04

**Authors:** Haider Alidrisi

**Affiliations:** Medicine, University of Basrah, Basrah, Basra Governorate, Iraq

Thanks for your valuable comments.

 1. The study included only transfusion-dependent BTH, we correct that in the revised version.

 2. Chief complaints frequencies were presented as presentations in Figure 1.

 3. We changed the wording from "prevalence" to "observed frequency and proportion "

 4. In our cohort, 181 patients had a single endocrine dysfunction, 38 had two dysfunctions, and only 11 had three or more dysfunctions. We agree that comparing their clinical presentations could be informative. However, the small and uneven sizes of the multiple-dysfunction groups, together with the numerous possible combinations of individual endocrine disorders, would produce sparse cells and unstable estimates. A formal comparison would therefore have limited statistical power and a substantial risk of spurious findings or overinterpretation. For this reason, we did not undertake additional inferential subgroup analyses or add further tables. We have instead expanded the Discussion to clarify that coexisting dysfunctions may reflect the shared, cumulative effects of iron overload, chronic hypoxia, oxidative stress, and duration or adequacy of transfusion and chelation therapy on several endocrine organs. Because the study did not assess iron-overload indices or chelation adequacy in relation to these combinations, the reasons for simultaneous double or triple dysfunctions cannot be established from our data. We have acknowledged this limitation and recommend that larger prospective studies evaluate the clinical phenotype and determinants of multiple endocrine dysfunctions.

# Reviewer report: Reviewer response for version 1

**DOI:** 10.5256/f1000research.201773.r504418

**Published:** 2026-08-06

**Authors:** Ahmed Ghdhban Al-Ziaydi

**Affiliations:** 1 University of Al-Qadisiyah, Al Diwaniyah, Iraq

Based on the study, the conclusions are generally supported by the reported results.

 Evaluation

 Strengths

 The results demonstrate that endocrine dysfunctions (EDs) were identified in the study population, with an overall prevalence of 16.8%.

 The most common endocrine disorders reported were growth hormone deficiency (6.0%), diabetes mellitus (4.9%), hypogonadism (4.5%), and hypothyroidism (3.2%), which directly supports the statement that these were the commonest EDs.

 The recommendation for regular and timely surveillance using clinical and hormonal assessment is reasonable because multiple endocrine abnormalities were identified among patients evaluated.

 Minor Concerns

 The study included only patients referred for endocrine-related complaints rather than all patients with beta-thalassemia major. Therefore, the reported prevalence may not represent the true prevalence in the entire BTH population and is subject to referral (selection) bias.

 The conclusion states that "EDs were prevalent in patients with BTH." Given that only 16.8% of the referred cohort was diagnosed with endocrine dysfunction and the study is retrospective and single-center, this wording may overgeneralize the findings. A more precise statement would acknowledge that the findings apply to this referral cohort.

 The recommendation for routine surveillance is clinically reasonable but is not directly evaluated by this retrospective study. The study demonstrates the occurrence of endocrine disorders but does not compare different surveillance strategies or show that earlier screening improves outcomes.

 Suggested Reviewer Comment

 The conclusions are generally supported by the presented results. The data support that growth hormone deficiency, diabetes mellitus, hypogonadism, and hypothyroidism were the most frequently identified endocrine disorders in this cohort. However, the conclusion should be interpreted with caution because the study included only patients referred for endocrine evaluation, introducing potential referral bias and limiting the generalizability of the reported prevalence to all patients with beta-thalassemia major. The recommendation for regular endocrine surveillance is reasonable but should be presented as a clinical implication rather than a direct finding of the study.

 Overall assessment: Yes – adequately supported, but the conclusions should be slightly tempered to reflect the retrospective, single-center, referral-based design.

Is the work clearly and accurately presented and does it cite the current literature?

Yes

If applicable, is the statistical analysis and its interpretation appropriate?

Yes

Are all the source data underlying the results available to ensure full reproducibility?

Yes

Is the study design appropriate and is the work technically sound?

Yes

Are the conclusions drawn adequately supported by the results?

Partly

Are sufficient details of methods and analysis provided to allow replication by others?

Partly

Reviewer Expertise:

Medical Biochemistry, Cancer cell, cell lines,

I confirm that I have read this submission and believe that I have an appropriate level of expertise to confirm that it is of an acceptable scientific standard.

# Response

**Published:** 2026-09-04

**Authors:** Haider Alidrisi

**Affiliations:** Medicine, University of Basrah, Basrah, Basra Governorate, Iraq

Thanks a lot for your valuable comments.

 We agree with suggestions to use a more concise wording for results of study based on the study design, and referral strategy. We revise our result data descriptions from "prevalence" to "observed frequency and proportions".

 In considering your comment about survielance strategy for patients with BTH for EDs, we agree with your comments that neither the study design nor finding support that as a conclusion. So we changed it to a recommendation.
